# MRI4ALL: A Week-Long Hackathon for the Development of an Open-Source Ultra-Low-Field MRI System

**DOI:** 10.1002/jmri.29771

**Published:** 2025-03-24

**Authors:** Kai Tobias Block, Chengtong Zhang, Vito Ciancia, Clarissa Cooley, Sairam Geethanath, Jason Stockmann, George Verghese, Leeor Alon

**Affiliations:** 1 Bernard and Irene Schwartz Center for Biomedical Imaging, Department of Radiology, NYU Grossman School of Medicine, New York, New York, USA; 2 Center for Advanced Imaging Innovation and Research (CAI2R), Department of Radiology, NYU Grossman School of Medicine, New York, New York, USA; 3 Department of Chemistry, New York University, New York, New York, USA; 4 LaGuardia Studio, New York University, New York, New York, USA; 5 A. A. Martinos Center for Biomedical Imaging, Boston, Massachusetts, USA; 6 Accessible MR Laboratory, Biomedical Engineering and Imaging Institute, Icahn School of Medicine at Mt. Sinai, New York, New York, USA; 7 Accessible MR Laboratory, Division of Cancer Imaging Research, Russell H. Morgan Department of Radiology and Radiological Science, Johns Hopkins University School of Medicine, Baltimore, Maryland, USA

**Keywords:** hackathon, low-field MRI, open-source, reproducible research

## Abstract

The goal of the MRI4ALL hackathon, which took place in October 2023, was to develop a functional low-field MRI scanner in just one week and to release all created source code and resources as open-source packages. The event was attended by 52 participants from 16 institutions who assembled the scanner on the last day of the hackathon. The scanner’s magnetic B_0_ field with a strength of 43 mT and a target field-of-view size of 11 cm^3^ was created with a Halbach array made from 990 N40UH permanent magnets, held in place using 3D printed ring formers. Gradient coils were fabricated by gluing enameled copper wire onto 3D printed holders with imprinted wire patterns. A solenoid coil for RF transmission and reception was built by winding 20 turns of Litz wire around a 3D printed cylinder. A Red Pitaya FPGA prototyping board running the MaRCoS framework was used to control the scanner components, and a GPA-FHDO amplifier board was used to drive the gradients. To simplify the scanner’s operation, console software with an intuitive graphical user interface was developed in Python using the PyPulseq package for sequence calculations. Furthermore, the scanner was equipped with a cooling system, as well as options for passive and active shimming. After resolving several technical issues that arose during the assembly, the scanner is now able to acquire MR images with different sequences. While not suitable for real-world clinical applications, it can be utilized for educational purposes or as a low-cost prototyping platform. Moreover, it may serve as a reference design for future MRI development projects. All source code and resources are available on the project website mri4all.org, allowing other groups to replicate the scanner.

**Evidence Level:**

n/a

**Technical Efficacy:**

Stage 1.

## 1 | Introduction

Clinical MRI scanners are highly complex systems and notoriously difficult to build, particularly due to the use of superconducting magnets for generating the main magnetic field. At present, only a handful of companies have the technical expertise and supply chains necessary to manufacture state-of-the-art scanners, which limits broad access to MRI hardware and development tools. For example, developing novel pulse sequences or reconstruction algorithms requires access to proprietary development kits, which are typically only available to select customers under research agreements that impose restrictions on the use and distribution. Moreover, detailed information about hardware components is usually kept undisclosed, rendering the scanner hardware largely a “black box.” As a result, publicly available information on the construction of MRI systems is scarce, and active participation in the advancement of scanner technology is limited to only a small group of experts. This lack of accessibility and transparency inevitably hampers innovation within the field.

Recently, however, multiple groups have presented novel ultra-low-field MRI designs that utilize permanent magnets and eliminate the need for superconducting coils [1–3]. While less powerful, these scanners can be manufactured at a substantially lower cost and without extensive engineering resources. Motivated by this recent progress, our group organized a hackathon event in October 2023, entitled “MRI4ALL” to answer the question whether it would be possible to construct a functional ultra-low-field MRI scanner in just one week. The event was attended by 52 researchers from 16 different institutions, who assembled the “Zeugmatron Z1” scanner on the last day of the hackathon. All created resources, including source code and hardware designs, were publicly shared as open-source packages after the event [4]. Although not suited for real-world clinical applications, the developed scanner generates MR images and is now used for student education and prototyping work. Additionally, it can serve as a reference design for future MRI development projects. The purpose of this paper is to summarize the experience from the hackathon, provide guidance to other groups intending to host similar events, and describe the technical details of the constructed low-field scanner.

## 2 | Materials and Methods

### 2.1 | Preparation and Planning

The hackathon was planned carefully in advance to ensure a successful outcome within the short event duration. Preparations began approximately 6 months before the event. After securing funding, an organizing team was formed with team leaders for the subprojects (1) magnet, (2) gradient system, (3) RF chain, (4) software, and (5) 3D design. The group met weekly to discuss design considerations and required procurements. Moreover, contingency plans were developed to address potential failure or damage to essential components, as ordering spare parts during the hackathon would not have been feasible.

A public website was created with an application form for interested participants. To promote the event, “Imaging the Unknown” was chosen as the hackathon motto, and it was pitched that a “mystery phantom” will be presented at the beginning of the hackathon and that the goal will be to reveal the inside of the phantom using the developed MRI scanner. Announcements were distributed via email and social-media channels.

### 2.2 | Main Magnet and Scanner Housing

**FIGURE 1 |:**
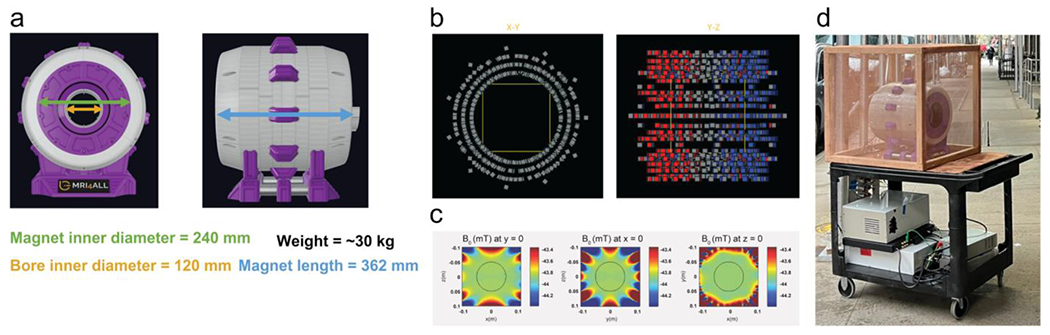
(a) CAD model of the scanner housing with dimensions. (b) Arrangement of the N40UH magnets into a Halbach array with multiple magnet rings and layers (color indicates the polarity). (c) Simulated field map of the Halbach array. (d) Assembled scanner with RF cage and electronic components on a movable utility cart.

The magnetic B_0_ field of the scanner was created with a Halbach array [5] of N40UH permanent magnets, which were arranged in multiple rings (Figure 1b). N40UH magnets were specifically chosen because of the high resistance to demagnetization. A relatively small magnet size of 12.7 × 12.7 × 12.7 mm^3^ was selected to reduce the likelihood of accidental injuries while handling the magnets. To calculate the exact placement of the N40UH magnets, a software program was written in Python using the Magpylib [6] and pymoo [7] packages. Magpylib is an open-source library that allows calculation of the resulting magnetic field from a given set of magnets. pymoo is an open-source library with numerical optimization tools, including a genetic optimization algorithm. By combining both packages, the optimal placement of the N40UH magnets with the highest field homogeneity was derived under given design constraints, which included a count of 990 individual magnets, a minimum inner diameter of 26.54 cm, a total length of 35.2 cm, and an outer diameter of 39.05 cm. The optimization varied the radius of each magnet layer (three total layers), the placement of each ring, and an angular offset between each ring along the *Z* axis. The estimated field strength was 43 mT for a target field-of-view (FOV) size of 11 cm^3^.

3D-printed ring formers with cubic cutouts were used for holding the magnets in place, which were designed by importing the calculated magnet arrangement into a CAD software (Fusion 360, Autodesk, USA). Polarity markings were added next to each cutout to avoid accidental misalignment of the magnet polarity during the assembly. Due to the size of the scanner, it was necessary to divide the ring formers into multiple smaller parts that could be fabricated with the available 3D printers. After populating the ring formers with the N40UH magnets, the parts were combined into 12 magnet rings and fastened together with brass bolts. To ensure a secure fit of the magnets and prevent bending of the parts from the magnetic forces, all components were made from polycarbonate.

Using the same CAD software, components for the scanner housing were designed (Figure 1a), including a solid stand to support the weight of the magnet (~30 kg) and mounts for the inserts described in the following sections. The complete 3D model is available for download in the GitHub repository [8]. Because 3D printing is a relatively slow process, the fabrication of most parts was done ahead of the hackathon and took approximately 10 days in total.

In addition to the scanner parts, the mystery phantom was designed and printed. The phantom, a non-transparent sphere with a diameter of 10 cm and a secret embedded message, was subsequently filled with a NiSO_4_-doped water solution and sealed (Figure 6b).

### 2.3 | Field Mapping and Shimming

**FIGURE 2 |:**
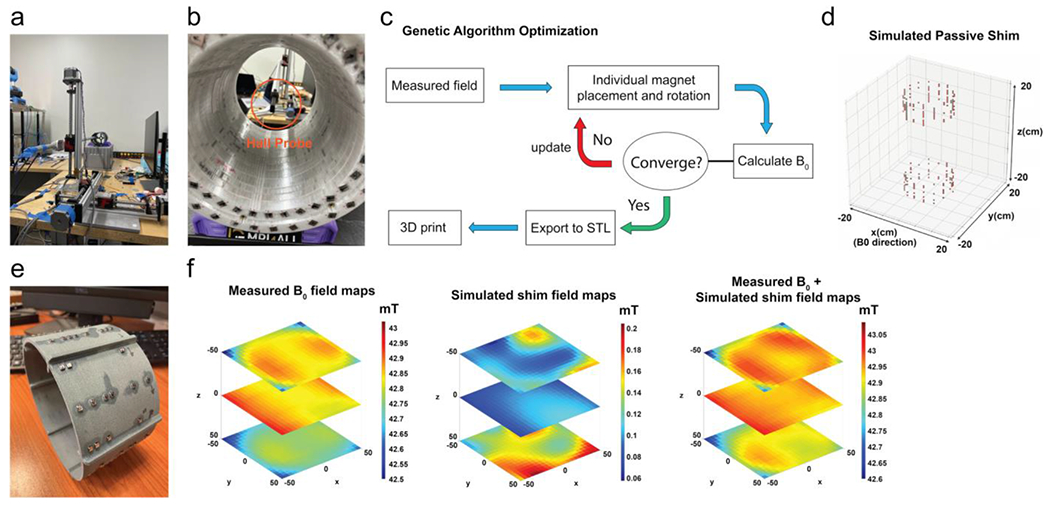
(a) Developed field-mapping robot, built from three connected linear CNC stages and a Hall probe. (b) The Hall probe was placed in the magnet and sequentially moved to different locations to create a field map. The measured field strength was stored each time. (c) Genetic optimization algorithm used to calculate the placement of the shim magnets from the field map. (d) Calculated placement of the shim magnets. (e) 3D printed holder with shim magnets. (f) Measured field map of the magnet (left), simulated field of the shim magnets (middle), and resulting field when adding the passive shim to the magnet (right).

To measure the homogeneity of the magnetic B_0_ field, a field-mapping robot was designed, consisting of a 3-axis Hall probe (THM1176-MF, Metrolab, Switzerland) and three connected CNC linear stage actuators for moving the probe in the *X, Y*, and *Z* directions (Figure 2a,b). The robot was controlled with an Arduino Uno Rev3 microcontroller board and a grblShield extension board with drivers for the three stepper motors of the actuators. The Hall probe was attached to one of the actuators using a hollow aluminum tube (inner diameter 10 mm) and custom aluminum brackets. Using control software developed during the hackathon, spatial B_0_ field maps could be measured and exported [9].

The field maps were utilized for calculating the placement of small shim magnets to compensate for construction inaccuracies and improve the B_0_ homogeneity (“passive shim”). Smaller N40UH magnets with a size of 3 × 3 × 3 mm^3^ were selected for this purpose. The shim magnets were attached to 3D-printed holders placed on both sides of the RF coil (Figure 2e). The holders were pre-designed in the CAD software and adjusted during the hackathon based on the field maps measured after the assembly of the magnet. The placement of the shim magnets was calculated using Magpylib and the genetic optimization algorithm, confining the radius to 66.5 mm, the number of rings to 22, the number of magnets per ring to 25, while fixing the holder position at 4 and 23.5 cm from the front of the magnet. The program then estimated the optimal ring positions along the bore of the main magnet, their rotations around the *z*-axis, and a binary decision on whether a magnet in a particular ring is needed or not [10].

In addition to the passive shim, the scanner was also prepared with an active shim option, consisting of 30 hand-wound coils with 15 turns and a diameter of 35 mm, mounted around an acrylic tube with an outer diameter of 152 mm. The tube was placed between the gradient coils and RF coil, allowing improvement of the B_0_ homogeneity by adjustment of the currents in each shim coil based on a measured field map. To prevent interference with the RF signal, an RF shield made from copper foil was placed on the inner side of the acrylic tube.

### 2.4 | Gradient Coils and Amplifiers

**FIGURE 3 |:**
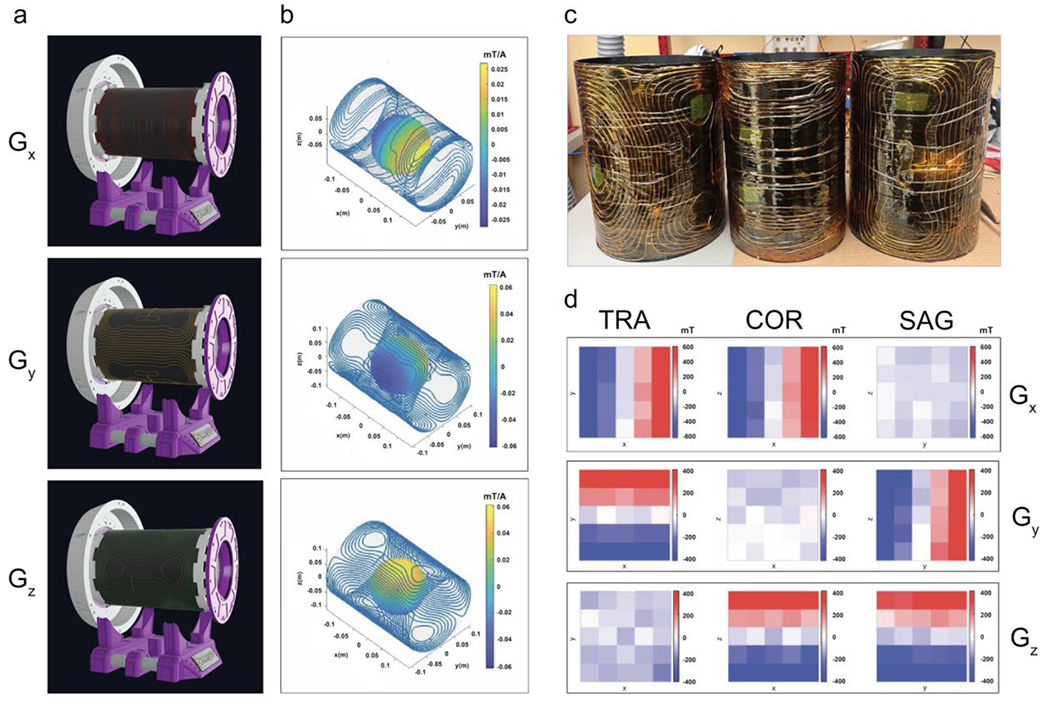
(a) CAD renderings of the 3D printed holders for the three gradient coils. (b) Simulated wire patterns of the gradient coils. (c) Fabricated gradient coils. (d) Magnetic fields created by the X, Y, and Z gradient coils, measured using the field mapping robot.

Wire patterns for the three gradient coils were calculated with the CoilGen open-source package [11] using a target FOV of 14 cm. The calculated patterns (Figure 3b) were imported into the CAD software. The coil windings were placed on the surfaces of three nestable cylinders, and a subtraction operation was applied to create 3D formers with grooves for attaching enameled 15AWG copper wire (Figure 3a).

The open-source “GPA-FHDO” gradient amplifier [12] was used to drive the gradient coils, providing 4 channels with current pulses of up to 10 A that are controllable via the Serial Peripheral Interface (SPI) communication bus (only 3 channels were used). A switching DC power supply (TekPower TP1560E) was set to 15 V and utilized to power the gradient amplifier. The gradient signals were filtered by connecting both ends of each gradient coil to filtering circuits made from a DC feedthrough capacitor (Schaffner FN7563-63-M6) and a high-current power inductor (CODACA CPER3231-101MC), which were attached to an earthed aluminum plate.

Because the gradient coils can generate heat during longer scans, and because permanent magnets are sensitive to temperature changes, a water-cooling insert was added between the magnet and gradient system. The cooling system was constructed from a 3D-printed holder for a silicon tube with a 1/4″ outer diameter, which was filled with a cooling liquid, and a heat exchanger originally intended for gaming computers (Pacific R360, Thermaltake Technology, Taiwan).

### 2.5 | RF Coil and Amplifiers

A single-channel solenoid coil [13] was designed for RF transmission and reception. The coil was built by winding 20 turns of Litz wire around a 3D-printed cylinder with a 66.5 mm outer radius and grooves for guiding the wire. It was tuned to an operating frequency of 1.825 MHz. Non-magnetic capacitors (American Technical Ceramics, 2500V, C series) were inserted at three locations of the Litz wire to distribute the electric field. A matching network was simulated with RFSim99, a freeware software for RF circuit design [14], and used to match the coil impedance to 50 Ω.

**FIGURE 4 |:**
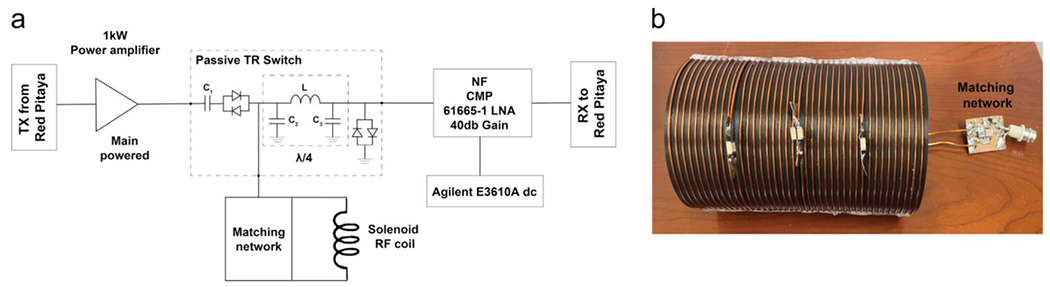
(a) Scheme of the scanner’s RF chain (the components of the T/R switch used *L* = 4.3 μH, C1 = C2 = 1.8 nF). (b) Solenoid RF coil made from 40 turns of copper wire around a 3D printed holder and the matching network with a BNC connector.

While it was originally planned to use a custom-built RF power amplifier (RFPA) created during the hackathon from an amateur-radio 1 kW LDMOS amplifier kit, an existing commercial RFPA was utilized instead to simplify the initial scanner setup (BT00250-AlphaS, Tomco Technologies, Australia). To allow for transmitting and receiving with the same RF coil, a passive T/R switch was designed and placed between the RF power amplifier and solenoid coil. On the receive side, a low-noise 40 dB preamp (CMP61665-1, NF Corporation, Japan) was integrated to amplify the signal, powered by a 15 V DC power supply (E3610A, Agilent Technologies, USA). A scheme of the complete RF chain is shown in Figure 4a.

### 2.6 | Component Control

**FIGURE 5 |:**
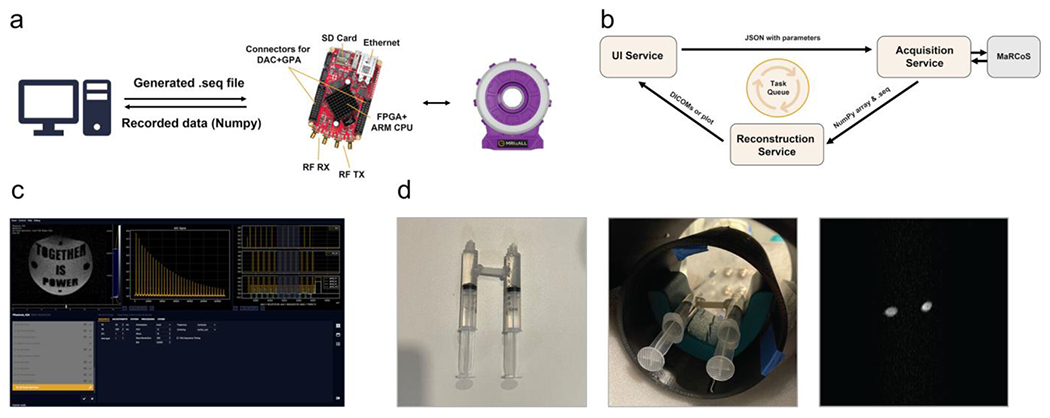
(a) Scheme of the scanner control using a Red Pitaya board. (b) Architecture of the developed console software. (c) User interface of the console software. (d) First image obtained by the scanner of a simple phantom made from two water-filled syringes.

A Red Pitaya SDRlab 122-16 prototyping board [15] was used to control the scanner components, which is based on a Xilinx Zynq 7020 field programmable gate array (FPGA) and capable of processing signals up to 60 MHz. The board provides two output (DAC) channels, two input (ADC) channels, and an SPI bus for connecting additional hardware, which was used to control the channels of the GPA-FHDA gradient amplifier. Furthermore, the board has an ethernet port for connecting a host computer and installing custom firmware. Red Pitaya boards have been used in multiple prior MRI development projects due to the relatively low price [16]. For the MRI4ALL scanner, the open-source MaRCoS framework was utilized [17], which was previously developed for Red-Pitaya-based MRI scanners. It includes a custom firmware image and a Python interface for executing sequence instructions from the host computer and retrieving the acquired data. It was combined with the FLOCRA-Pulseq interpreter [18], which converts sequences from the Pulseq format [19] into the MaRCoS instruction syntax. This enabled the use of the open-source PyPulseq package [20] for sequence calculations and running them on the Red Pitaya board via the MaRCoS framework. Acquired data is returned in the NumPy format (Figure 5a).

### 2.7 | Console Software

To simplify the scanner usage, console software with an intuitive graphical user interface (GUI) was developed, similar to those of commercial MRI systems. The GUI allows configuring exam protocols, performing scans, and viewing results (Figure 5c and Video S1). Only open-source libraries and tools were utilized for the development. Ubuntu 22.04 was used as the operating system, and Python was selected as the primary programming language because it is widely accepted and offers many libraries for scientific computing. The GUI was realized with PyQt5 [21], which was deemed most suitable for rapid prototyping. Qt Creator was used for the interface design [22], and the matplotlib [23] and PyQtGraph [24] libraries were integrated for visualizing results.

A standardized development environment was created, including a provisioning script for automated installation on Intel-based computers using VirtualBox [25] and Vagrant [26], which instantiates a new virtual machine, clones the code repository, and installs all required dependencies. Visual Studio Code was selected as the editor [27]. When installed on the host computer, it connects to the virtual machine over SSH for code editing, while the authentication for GitHub code submissions is managed on the host. Therefore, new instances of the development environment can be created in just minutes without user interaction. Instructions were shared with the participants prior to the hackathon to avoid delays on the first day of the event.

At the code level, the software was divided into three decoupled service processes to provide a responsive user experience (Figure 5b): 1. UI, which provides all frontend functionality for planning scans and viewing results; 2. ACQ, which executes scheduled scans using MaRCoS and stores the raw data; and 3. RECO, which reconstructs images from the raw data and triggers the visualization in the UI. This modular architecture allows users to configure new scan protocols or view results while the scanner is busy running a sequence. The services interact through a folder-based task queuing system. In addition, the services can communicate through an inter-process communication (IPC) mechanism to trigger adjustment dialogs or status updates.

Folder-based task queuing has the advantage that each service can be developed and tested independently by injecting test tasks into the queuing path. This allowed the development teams to work in parallel during the hackathon. Tasks are stored in separate subfolders and contain a file in JSON format [28] that describes the requested sequence type, scan parameters, adjustment and reconstruction settings, scanner information, and a list of results, which is populated during a scan. Moreover, scan tasks contain defined subfolders, into which the services are expected to write generated files, such as acquired raw data or reconstructed images. The ACQ and RECO services have separate queues and process one task at a time. Completed and failed tasks are archived with all intermediate files and can be easily inspected for debugging.

Custom sequences can be integrated into the platform by deriving a Python class from the sequence parent class, which acts as an application interface and allows enumerating installed sequences. The interface includes entry functions for loading and saving parameters, showing controls in the UI, performing calculations (typically using Pulseq), and executing the sequence. Therefore, low effort is needed for adding new sequences because common tasks, such as state or protocol management, are covered by the platform. At the same time, high flexibility is retained, allowing sequences to show arbitrary UI controls or to employ alternative libraries for sequence generation. Various sequences were implemented during the hackathon, including adjustment sequences (e.g., for frequency adjustment), debugging sequences (e.g., for testing the gradients), and basic imaging sequences.

A basic FFT-based reconstruction for 2D and 3D scans with a flexible k-space acquisition order was integrated as the default algorithm into the RECO service. Additional reconstruction algorithms can be integrated by adding handlers to the RECO service. Custom algorithms must read the raw data in the NumPy format [29] and write out DICOM files into a defined subfolder of the task. Reconstruction and scan parameters can be read from the JSON file in the task folder. Algorithms can create either one image series or multiple series per scan. Moreover, algorithms can specify which image series (or plots) should be automatically loaded into the GUI.

Because the platform has been written entirely in Python, development times were short without the need for compiling the code. As a further advantage, machine-learning techniques, such as deep-learning-based reconstruction or denoising, can be easily integrated into future versions by importing the PyTorch library [30]. The code and software documentation are provided on GitHub [31].

## 3 | Results

### 3.1 | Experience and Timeline

Seventy-four researchers applied for the hackathon. Due to space constraints, it was necessary to limit the number of participants to 52. Applicants were selected based on their technical background and experience level, aiming for an equal mix of experienced and junior researchers as well as a balance in gender distribution. Participants were assigned to project teams based on their preferences listed during the application.

The participants met on the evening before the hackathon for an informal get-together. The event started on Monday at 9 am with a kick-off meeting during which the teams were introduced and the plan for the week was discussed. Furthermore, a naming contest for the scanner was held. It was decided to call the scanner “Zeugmatron Z1” in reference to the term “Zeugmatography” that Paul Lauterbur proposed in his seminal paper from 1973 [32]. Afterwards, project work began in the different teams, and participants worked as late as they liked to. The other hackathon days started with a breakfast meeting at 9 am where the teams reported on the progress and challenges, followed by project work until the evening. Work paused on Wednesday and Thursday for a scientific conference, which allowed printing additional scanner parts. The hackathon concluded on Saturday evening with the scanner assembly and a farewell gathering.

### 3.2 | Magnet Construction

The team measured and marked the polarity of all 990 N40UH magnets using Hall sensors and magnet-polarity detectors. Afterwards, the ring formers were populated, and the magnet rings were combined. The rings were bolted together starting from the lower end of the magnet. After the completion of the magnet, the field-mapping robot was used to measure the homogeneity, which revealed a quite significant deviation from the simulation (5987 vs. 740 ppm over a 10 cm DSV). This difference was caused by minor 3D-printing inaccuracies and magnet forces, leading to gaps of up to 2 mm between segments of the rings. Therefore, it was decided to disassemble the magnet again and glue the rings together under force using epoxy, which improved the homogeneity to 3642 ppm. The residual difference between the simulated and measured field might also stem from impurities of the permanent magnets and demagnetization effects.

The measured field map was then used to calculate the placement of the shim magnets. After printing the shim sleeves and attaching the small N40UH magnets with superglue, the measured field homogeneity was reduced to 2831 ppm.

### 3.3 | Coil Fabrication

The gradient coils were fabricated by bending the enameled copper wire segment by segment along the grooves of the 3D-printed holders and fixing it with superglue. Clamps were used to hold the wire in place while the glue was drying. Next, the coils were covered with epoxy to avoid movement from torquing during gradient switching. The coils were then wrapped in a layer of Kapton tape to prevent the epoxy from bonding with adjacent components. As the last step, the three coils were assembled into a single gradient insert.

The inductance of each gradient coil was measured with an automatic LCR meter. The measured inductance values (49, 73, and 101 μH) were nearly identical to the simulations (48, 72, and 97 μH) for Gx, Gy, and Gz. The coil resistance was measured via the voltage across the coil with a DC power supply providing a current of 1 Amp, which showed slightly larger resistance (0.36, 0.38, and 0.45 Ω) than in the simulations (0.23, 0.28, and 0.38 Ω). Last, the magnetic field created by each coil was measured using the field-mapping robot for a 10 cm FOV in each direction, from which the gradient strengths were calculated by first-order spherical harmonic fitting. The measured values (0.39, 0.88, 0.85 mT/m/A) were similar to the CoilGen simulations (0.35, 0.92, 0.86 mT/m/A).

The solenoid RF coil was assembled by winding 20 turns of Litz wire along the grooves of the prefabricated holder and securing it with hot glue. The coil was tuned to a resonance frequency of 1.825 MHz using a network analyzer. The matching network was built and soldered to the coil. A BNC connector was soldered to the other end of the matching network, so that the RF coil can be easily exchanged. A second solenoid coil with a smaller radius of 47.5 mm and 40 turns was assembled as a backup using regular copper wire (Figure 4b).

### 3.4 | Scanner Assembly

The magnet was placed on the upper level of a movable utility cart. Electronic components, such as amplifiers and power supplies, were placed on the lower level of the cart. In the first step, the cooling system was inserted into the magnet, and the silicon tubing was connected to the heat exchanger on the lower level. Next, the gradient system was inserted, which required slight force due to the increased radius from the epoxy. The aluminum plate with the gradient filter circuits was bolted against the frame of the cart, and the gradient wires and GPA outputs were connected. The acrylic tube with the active-shim loops and RF shield was inserted next. Finally, the RF coil with the passive-shim sleeves was inserted, and the mystery phantom was placed in the center of the RF coil. The Red Pitaya board was connected to the RF chain and GPA, as well as to a Dell OptiPlex 7090 MICRO host computer via ethernet. The console software was installed on the host computer. The fully assembled scanner can be seen in Figure 1d. Additional pictures are available online [33].

### 3.5 | Troubleshooting

Unfortunately, the scanner malfunctioned on the final evening of the hackathon. Troubleshooting efforts on the subsequent days identified three main issues: 1. A loose connection was found in the RF coil, which was resoldered. 2. The distance between the RF shield and RF coil was found to be too small, resulting in cancellation of the RF fields and very low received signal levels. This issue was resolved by temporarily removing the acrylic tube with the internal RF shield (including the shim sleeves, which are held by the acrylic tube). 3. Strong electromagnetic interference and noise contamination of the power lines were observed in the building, likely originating from nearby machine rooms with air-conditioning equipment and transformers. This problem was addressed by moving the scanner temporarily into an empty clinical scanner bay with RF shielding and by adding a power conditioner. Moreover, a removable RF cage was constructed from copper mesh (Figure 1d), allowing the scanner to be used in different locations.

### 3.6 | Phantom Scans

**FIGURE 6 |:**
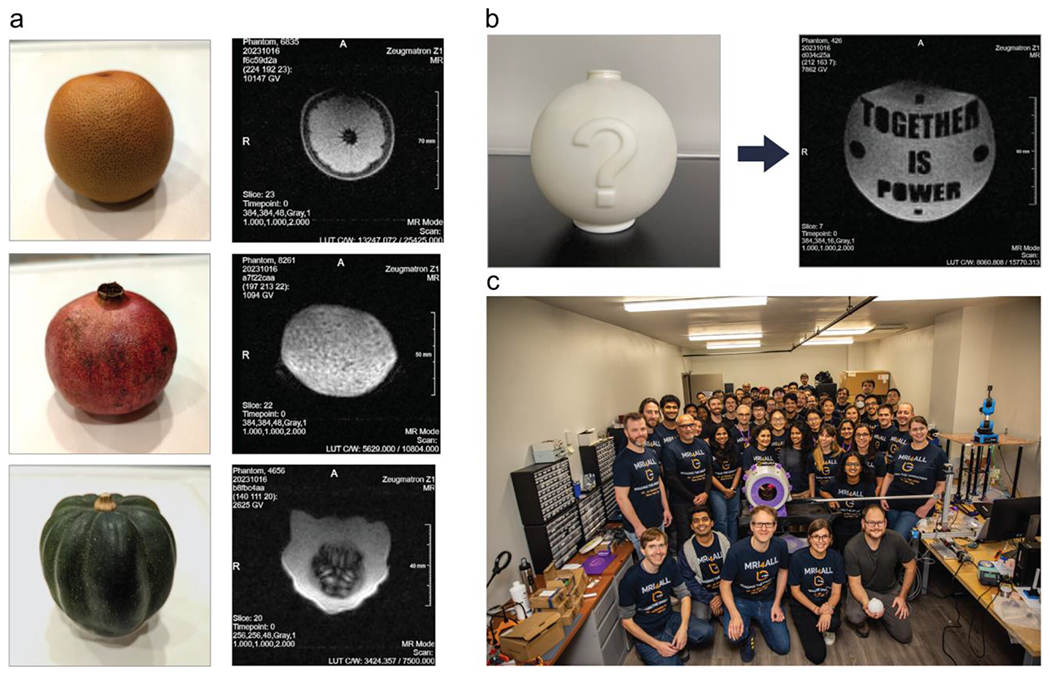
(a) Exemplary MR images obtained with the scanner of a grapefruit, pomegranate, and acorn squash. (b) The mystery phantom and a high-resolution MR image acquired with the scanner, revealing the secret message embedded in the phantom. (c) Group photo of the hackathon participants with the assembled scanner and field-mapping robot.

After addressing these issues, the scanner was able to receive clean, phase-stable spin echoes. As an initial test, 2D spin-echo images were acquired of a simple phantom made from two water-filled syringes (Figure 5d). Next, high-resolution images of the mystery phantom were acquired, which revealed the secret message embedded in the phantom (Figure 6b). For this purpose, a 3D (single) spin-echo sequence was used (TE 20 ms, TR 500 ms, 16 slices, 384 pixels base resolution, TA 51:14). Finally, scans of different fruits and vegetables were acquired with the 3D spin-echo sequence, which are shown in Figure 6a.

## 4 | Discussion

To our knowledge, the MRI4ALL hackathon was the first community event where a group of scientists from different institutions came together to build a complete open-source MRI scanner. While the Zeugmatron Z1 scanner is of educational nature and not suited for real-world medical applications, many components and concepts are similar to those used in clinical MRI scanners. Therefore, the hackathon offered an excellent learning experience for the participants. All information about the scanner has been publicly shared on the project website, including a detailed component list with cost estimates for the individual scanner parts [33]. The total material costs were around $15,000 (excluding the commercial RFPA), of which approximately $8000 were spent on 3D printing due to the use of high-quality polycarbonate filaments for withstanding the magnet forces. However, this expense could possibly be reduced by utilizing more affordable filaments.

Because of the relatively low price and the use of 3D printing, other institutions can reproduce the scanner and employ it in educational classes on MRI, avoiding the need for expensive scan time on clinical scanners. For example, the scanner can be utilized for practical exercises on RF coil design, the development of reconstruction algorithms, or sequence programming. For the latter, the use of Pulseq is attractive because the gained knowledge can be directly applied to clinical scanners. Moreover, since the development environment can be freely installed on laptops, students can use the console software to conduct exercises on image reconstruction and signal processing using data previously acquired with the scanner. The scanner can also be used as a low-risk prototyping platform for evaluating new imaging concepts that require hardware modifications, which cannot be easily realized on clinically utilized scanners.

### 4.1 | Improvements

The examples in Figure 6 show that the image quality produced by the scanner is limited and significantly lower than that of regular MRI systems. In particular, strong distortions can be seen which result from the inhomogeneity of the B_0_ field. While the focus of the hackathon was on the educational aspect and not on creating a high-quality scanner, various enhancements could be made to the scanner for improving the quality. All images shown in this article were acquired without passive and active shimming (due to removal of the inner RF shield). Installing the passive shim should improve the homogeneity by around 25%, as already validated with the field-mapping robot. Moreover, simulations have suggested that the active-shim array would improve the homogeneity drastically. It would also allow for object-specific dynamic shimming. This may enable using SNR-effective sequence types such as 3D balanced SSFP, which have been tested on the scanner but produced severe banding artifacts due to the currently high inhomogeneity.

Another limitation of the scanner consists in the sensitivity to electromagnetic interference (EMI), which required the installation of an RF cage. Several groups have presented promising results using EMI cancellation techniques [34–36], which allow for the removal of the outer RF shield. Since the Red Pitaya board has two input channels, a reference signal from an EMI sensing coil could be acquired in parallel with the MRI signal and employed to subtract the EMI noise before image reconstruction. A prototype coil for EMI sensing has already been built during the hackathon, but there was insufficient time during the event to work on the integration of EMI cancellation.

A further limitation consists in the temperature sensitivity of the permanent magnets, which results in noticeable shifts of the B_0_ field strength depending on the room temperature. While adjustment sequences for automatic selection of the resonance frequency have been integrated into the console software, the RF coil has been tuned for a fixed resonance frequency. Thus, the achievable signal-to-noise ratio varies with the temperature of the environment. This problem may be addressed by integrating temperature sensors into the magnet, ideally between the magnet and the gradient coils, for monitoring temperature drifts and adjusting the cooling system to keep the magnet at a constant temperature. These ideas may be implemented during a sequel hackathon event.

## 5 | Conclusion

The MRI4ALL hackathon demonstrated that a functional MRI scanner can be built in a short amount of time when utilizing permanent magnets, 3D printing, and the collective efforts of a large, motivated team. The developed scanner is primarily of educational value and can be reproduced by other institutions based on the open-source packages and resources provided on the project website. It may help to democratize access to MRI development resources and enable a broader group of researchers to contribute to the advancement of MRI technology. Moreover, the amount of components developed during the event showed that the format of a hackathon, which frees up researchers from administrative tasks and provides protected time for technical work, can be a highly effective mechanism for creating resources of communal interest.

## Supplementary Material

Supp 1

Additional supporting information can be found online in the Supporting Information section.

## Abbreviations:

ADC: analog to digital converter
CAD: computer aided design
CNC: computer numerical control
DAC: digital to analog converter
DC: direct current
DSV: diameter of spherical volume
EMI: electromagnetic interference
FOV: field of view
FPGA: field programmable gate array
GPA: gradient power amplifier
GUI: graphical user interface
MRI: magnetic resonance imaging
N40UH: sintered ultra-high-temperature neodymium-iron-boron magnets
PPM: parts per million
RF: radiofrequency
RFPA: radiofrequency power amplifier
SPI: serial peripheral interface
SSH: secure shell
T/R: transmit/receive
